# Genome editing enables defense-yield balance in rice

**DOI:** 10.1007/s44154-023-00102-4

**Published:** 2023-07-07

**Authors:** Yiwen Deng, Zuhua He

**Affiliations:** National Key Laboratory of Plant Molecular Genetics, CAS Center for Excellence in Molecular Plant Sciences, Institute of Plant Physiology and Ecology, Chinese Academy of Sciences, Shanghai, China

**Keywords:** Lesion mimic mutant, Rice, Broad-spectrum resistance, Phospholipids, Genome editing

## Abstract

This brief article highlights the key findings of the study conducted by Sha et al. (Nature, doi:10.1038/s41586-023-06205-2, 2023), focusing on the cloning of the *RBL1* gene from rice, which is associated with lesion mimic mutant (LMM) traits. The *RBL1* gene encodes a cytidine diphosphate diacylglycerol (CDP-DAG) synthase and plays a crucial role in regulating cell death and immunity by controlling phosphatidylinositol biosynthesis. The *rbl1* mutant shows autoimmunity with multi-pathogen resistance but with severe yield penalty. Using genome editing techniques, the research team successfully generated an elite allele of RBL1 that not only restores rice yield but also provides broad-spectrum resistance against both bacterial and fungal pathogens. These findings demonstrate the potential of utilizing genome editing to enhance crop productivity and pathogen resistance.

Global food demand is on the rise due to the growing world population, but crop production faces the persistent threats from various diseases. According to the Food and Agriculture Organization of the United Nations (FAO), fungal diseases alone account for 10–23% of annual global crop yield losses, equivalent to the food supply of 600–4,000 million people (Stukenbrock and Gurr 2023). Rice (*Oryza sativa*), a staple crop for more than half of the world’s population, is significantly reduced by major rice diseases including rice blast caused by *Magnaporthe oryzae* (*M. oryzae*) and rice false smut caused by *Ustilaginoidea virens* (*U. virens*). Rice false smut not only reduces grain yield but also contaminates grains with mycotoxins. Breeding disease-resistant varieties is a primary strategy for controlling crop diseases. However, pathogens constantly evolve, capable of overcoming host resistance within a few years. This process is exacerbated by global climate change and widespread monocultures, which accelerate pathogen adaption. Consequently, cloning and using durable broad-spectrum disease resistance genes with no or less yield penalties is considered the most effective and sustainable approach to control crop diseases (He et al. 2022). However, until now, only a limited number of such genes have been isolated from natural crop populations, including rice *Pigm* (Deng et al. 2017), *Bsr-d1* (Li et al. 2017a, b) and wheat *TaPsIPK1* (Wang et al. 2022). Therefore, it is urgent to explore additional sources of resistance to combat evolving pathogens effectively and ensure the long-term viability of crop production.

Lesion mimic mutants (LMMs) spontaneously develop necrotic lesions resembling hypersensitive responses triggered by resistance genes without being challenged by pathogens. LMMs usually confer more durable and broader-spectrum resistance than typical *R* genes (Li et al. 2019; Gao et al. 2021). LMMs encode proteins with diverse functions, including the regulation of gene transcription, protein translation and modification, metabolism, as well as vesicular trafficking. Research focused on these LMMs has significiantly contributed to our understanding of plant biology, particularly the plant immune system. However, utilization of LMM genes poses a technical challenges as they often confer disease resistance at the expense of reduced crop yield.

The paper by Sha et al. demonstrates a strategy to harness LMM genes while minimizing yield losses. The lesion mimic mutant *rbl1* was identified from a rice mutant collection, exhibiting strong autoimmunity responses and enhanced resistance to rice blast and bacterial blight caused by *Xanthomonas oryzae* pv. *oryzae* (*Xoo*) (Li et al. 2017a, b). The target gene *RBL1* encodes a CDP-DAG synthase (CDS1), responsible for catalyzing the synthesis of CDP-DAG using cytidine triphosphate (CTP) and phosphatidate. Similar to other LMMs, the *rbl1* mutant line exhibits enhanced disease resistance but suffers from a severe reduction in grain yield. To optimize the *RBL1* gene for disease resistance, the research team employed genome editing techniques to target multiple sites within its genic region. They found that a 12-bp deletion in the second exon of *RBL1*, named *RBL1*^*Δ12*^ allele, not only restored plant growth but also maintained broad-spectrum disease resistance against multiple strains of *M. oryzae*, *U. virens*, and *Xoo* strains. In a multi-location field trial, the *rbl1*^*Δ12*^ line did not compromise the yield and maintained robust resistance to blast. This *RBL1*^*Δ12*^ allele has an obvious agronomical value. It would be intriguing to investigate how RBL1^Δ12^ regulates the trade-off between disease resistance and grain yield in rice, which facilitating using it precisely in other crops. The reverse phenomenon to the one shown by Sha et al. has been reported in *R* genes cloned from natural populations, in which mutation in the *R* gene leads to the constitutive activation of the R protein, resulting into the lesion mimic phenotype (Zhou et al. 2019). It is plausible that *R* genes obtained from natural populations have undergone fine-tuning during the long process of evolution and domestication. The use of intensive genome editing techniques, as demonstrated by Wang et al. (2022) and Sha et al. (2023) enables the rapid achievement in a short time. With the rapid development of genome-editing technologies and the availability of many novel tools, such as long fragment knock-in, single-base substitutions, similar applications of similar approaches to other genes and crops are becoming increasingly feasible (Gao 2021; Wang and Doudna 2023). Given that RBL1 homologs are conserved in various crops, the editing of *RBL1* genes and evaluating the resulting gene variants in different crop species probbaly provide great potential for enhancing disease resistance.

The study provides mechanistic insights into the role of phospholipids in the interactions between *M. oryzae* and rice. The enzyme RBL1 is responsible for converting phosphatidic acid (PA) into CDP-DAG, which is a crucial metabolic intermediate in the biosynthesis of multiple phospholipids (Jennings and Epand 2020). The study by Sha et al. demonstrates a significant reduction in phosphatidylinositol (PI) and phosphatidylglycerol (PG) level in *rbl1* mutant, with the reduction of PI being identified as the key factor underlying the observed phenotype. Additionally, the study shows the reduction in membrane phosphatidylinositol 4,5-bisphosphate (PI(4,5)P_2_) in the *rbl1* mutant. Importantly, the study reveals the dynamic location of PI(4,5)P_2_ during early infection stages. PI(4,5)P_2_ is rapidly recruited to the infection site, encapsulating the hyphal tip, and subsequently becomes enriched in the biotrophic interfacial complex (BIC) and extra-invasive hyphal membrane (EIHM) (Simon et al. 2014) (Fig. 1). These cellular structures are likely critical for fungal infection, as the BIC serves as a gateway for the cytoplasmic effectors of *M. oryzae* to be secreted into the rice cell. These effectors are known to play critical roles in suppressing host immune responses, thereby facilitating successful infection (Oliveira-Garcia et al. 2023). The involvement of PI(4,5)P_2_ in this immnue process suggests that phosphatidylinositol phosphates (PIPs) may have a significant role in pathogen-host interactions and disease resistance across various pathosystems, although the underlying mechanism keeps unclear. Studies on powdery mildew infection have indicated that PI(4,5)P_2_ acts as a susceptibility factor (Qin et al. 2020), and another fungal pathogen *Colletotrichum higginsianum* modifies the PI(4,5)P_2_ levels in the EIHM to facilitate successful infection of host cells (Shimada et al. 2019). Additionally, plant virus hijacks PIP_2_ via its viral capsid protein to evade autophagic degradation by the host (Wang et al. 2023). Multiple genes involved in the biosynthesis of PI(4,5)P_2_ have been predicted in plants. However, their specific functions in rice immunity remains unexplored. In Arabidopsis, *pip5k1 pip5k2* mutants showed broad-spectrum disease resistance, further supporting the importance of PI(4,5)P_2_ in plant immunity (Qin et al 2020). Therefore, within the context of lipid metabolic pathways associated with rice immunity, other genes with functions related to *RBL1*, including those encoding phosphatidylinositol phosphate kinases (*PIPKs*) directly involved in PIPs biosynthesis, remain to be investigated. More generally, what is the role of *RBL1* in the established PAMP-triggered immunity (PTI) and effector-triggered immunity (ETI) immune system is still obscure. Additionally, phospholipids also play a significant role in the interaction between arbuscular mycorrhizal fungi and their host plants (Noack and Jaillais 2020). It would be worthy of investigating how RBL1 and related alterations in phospholipids impact the symbiotic process between crops and arbuscular mycorrhizal fungi.

Overall, this study presents a novel approach to harness the potential of LMMs by fine-tuning their metabolism using genome editing. This strategy offers a promising avenue for developing broad-spectrum resistance in crops. Additionally, the study highlights the significance of phospholipids in microbe-host interactions. Further insights into the role of phospholipids in the microbe-host interactions could lead to rational design of novel ways of crop protection, with developing disease-resistance crops and fungicides targeting these critical phospholipids and structures (Zhou et al. 2021), which should contribute to strengthening our understanding plant immunity as well as crop breeding (Fig. 1).

**Fig. 1:**
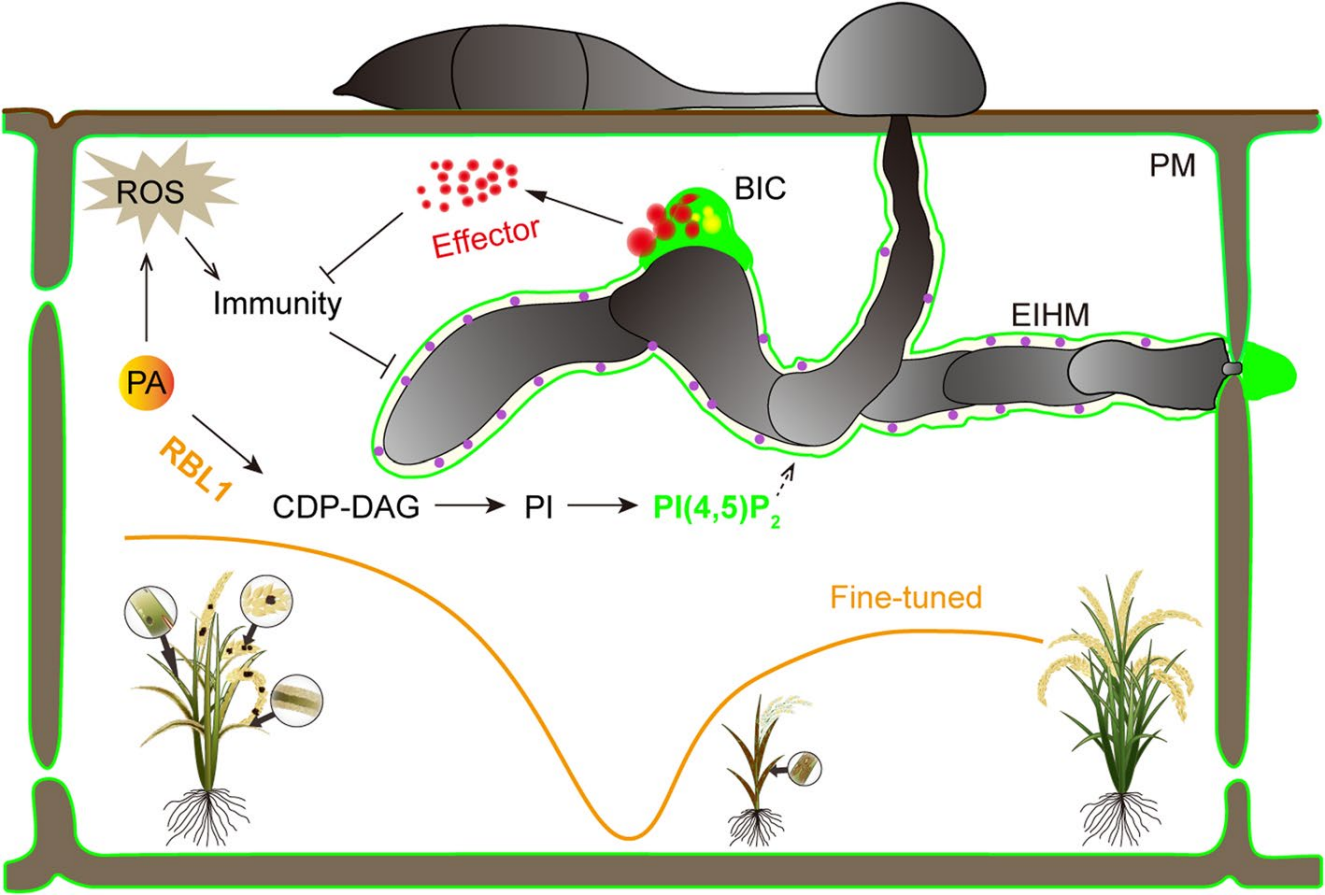
Role of phospholipids in *M. oryzae*-rice interactions. BIC, biotrophic interfacial complex; EIHM, extra-invasive hyphal membrane; PA, phosphatidic acid; PI, phosphatidylinositol; PI(4,5)P_2_, phosphatidylinositol 4,5-bisphosphate; PM, plasma membrane; ROS, reactive oxygen species

## Data Availability

Not applicable.
